# COVID-19: insights into virus–receptor interactions

**DOI:** 10.1186/s43556-021-00033-4

**Published:** 2021-04-10

**Authors:** Azadeh Sepahvandi, Maryam Ghaffari, Amir Hossein Bahmanpour, Fathollah Moztarzadeh, Payam Zarrintaj, Hasan Uludağ, Masoud Mozafari

**Affiliations:** 1 Department of Mechanical Engineering College of Engineering and Computing, University of South Carolina, 301 Main St, Columbia, SC 29208 USA; 2 Biomaterial Group, Faculty of Biomedical Engineering (Center of Excellence), Amirkabir University of Technology, Tehran, Iran; 3 School of Chemical Engineering, Oklahoma State University, 420 Engineering North, Stillwater, OK 74078 USA; 4 Department of Chemical and Material Engineering, Faculty of Engineering, University of Alberta, Edmonton, AB T6G 2V4 Canada; 5 Faculty of Pharmacy and Pharmaceutical Sciences, University of Alberta, Edmonton, AB T6G 2E1 Canada; 6 Department of Biomedical Engineering, Faculty of Medicine and Dentistry, University of Alberta, Edmonton, AB T6G 2R3 Canada; 7 Department of Tissue Engineering & Regenerative Medicine, Faculty of Advanced Technologies in Medicine, Iran University of Medical Sciences (IUMS), Tehran, Iran; 8 Currently at: Lunenfeld-Tanenbaum Research Institute, Mount Sinai Hospital, University of Toronto, Toronto, ON Canada

**Keywords:** COVID-19, Coronavirus, SARS-CoV-2, Thermodynamics, Virus-cell interactions, ACE2, Conformational changes, Glycosylation, Hydrophobicity

## Abstract

The recent outbreak of Coronavirus Disease 2019 (COVID-19) calls for rapid mobilization of scientists to probe and explore solutions to this deadly disease. A limited understanding of the high transmissibility of SARS-CoV-2 (Severe acute respiratory syndrome coronavirus 2) relative to other coronavirus strains guides a deeper investigation into the virus/receptor interactions. The cutting-edge studies in thermodynamic and kinetic properties of interactions such as protein-protein interplays have been reviewed in many modeling and analysis studies. Highlighting the thermodynamic assessments of biological interactions and emphasizing the boosted transmissibility of SARS-CoV-2 despite its high similarity in structure and sequence with other coronavirus strains is an important and highly valuable investigation that can lead scientists to discover analytical and fundamental approaches in studying virus’s interactions. Accordingly, we have attempted to describe the crucial factors such as conformational changes and hydrophobicity particularities that influence on thermodynamic potentials in the SARS-COV-2 S-protein adsorption process. Discussing the thermodynamic potentials and the kinetics of the SARS-CoV-2 S-protein in its interaction with the ACE2 receptors of the host cell is a fundamental approach that would be extremely valuable in designing candidate pharmaceutical agents or exploring alternative treatments.

## Introduction

Coronaviruses like SARS-CoV-2 enters and infect human cells through spike (S) glycoprotein binding to the cell membrane protein angiotensin-converting enzyme 2 (ACE2) [1, 2]. The ectodomain of S-protein comprises two functional subunits. An N-terminal subunit named S1 is responsible for binding to the host cell receptor and a C-terminal subunit named S2 that is responsible for the fusion of the virus with lipid membrane [3]. S1 subunit contains two subdomains: an N-terminal domain and the C-terminal domain, which is known as a receptor-binding domain (RBD) [4]. SARS-CoV-2 uses its RBD to directly bind to the peptidase domain (PD) of ACE2 [5]. More recently, scientists have used cryogenic electron microscopy (cryo-EM) to study the structure of the S-protein and ACE2 when it is bond to one of its typical ligands in the viral fusion [6]. S-protein is cleaved by host proteases at S1/S2 boundary and the called S2′ site located closely upstream of the S2. This cleavage facilitates membrane fusion via wide irreversible conformational changes. In SARS-CoV-2, the presence of a furin cleavage site at the S1/S2 boundary has identified [7]. By abrogation of this cleavage motif, the SARS-CoV-2 S-mediated entry into cells was affected [8]. Thus, different features of the cleavage site at SARS-CoV-2 can be considered as one of the factors influencing the high rate of the viral spreading and pathogenesis [9]. Kirchdoerfer et al. [10] studied the influence of stabilizing mutations of SARS-CoV S-protein on the conformational transitions of S-protein. They indicated that ACE2-binding or tyrosine cleavage at the S1/S2 boundary could not induce large conformation change in stabilizing mutation of SARS-CoV S-protein. Thus, a study on the furin-like cleavage that has not identified in other SARS-like CoVs would be the focus of studies to develop efficient anti-viral drugs [11]. For instance, a docking study suggested that heparan sulfate enhance the open conformation of the RBD so enhance the binding to ACE2 [12]. The structural studies showed that S-protein is also heavily glycosylated, possibly affecting the receptor binding kinetics. The structural dynamics of the S-protein is an intrinsic property and its features are strongly influenced by the overall chemical, physical and electrical charge of these carbohydrates [13].

The detailed aspects of the thermodynamics governing the relationship between the structure and function of macromolecules are often swamped in the molecular and biochemical events and crucial information on mechanisms of interactions are lost. The need for a clarification of thermodynamic events in biologic interactions has been recognized for a long time since the pioneering studies on the protein structures [14]. For many years, our understanding of thermodynamic events and kinetics of these interactions in biological systems have remained limited to macroscopic quantities accessible by experimental measurements [15, 16]. Recent advances in the visualization of nanoscale and dynamic molecular simulation have made it possible to access information at the atomic level and describe crucial events in individual binding sites, amino acid interactions, and protein folding [17, 18]. It is worth mentioning that CoV (coronavirus)-receptor interaction is a complex process unlikely to be regulated only by the binding energy of the S-protein with ACE2. Our goal is to characterize the thermodynamic potentials and kinetics of the SARS-CoV-2 S-protein in its interaction with ACE2 receptors of the host cell. In this regard, we focus on the structural and conformational differences between SARS-CoV (Severe acute respiratory syndrome coronavirus) and SARS-CoV-2 S-protein RBDs to discuss binding energy changes of S-protein and following thermodynamic potentials changing in the process of S-protein adsorption into ACE2. These assessments would be tremendously valuable in the design of candidate pharmaceutical agents exploring effective intervention in viral infection [19]. In the following sections, we will discuss about the basic thermodynamic parameters about virus-cell interactions, influential factors on binding thermodynamic potentials, various forms of entropy involved in binding free energy, effects of glycosylation sites of S-protein on thermodynamic principals, as well as the role of electrostatic interactions in virus-cell interaction enthalpy-entropy compensation in the virus-cell interactions.

## S-protein structure and its interaction with ACE2

**Fig. 1:**
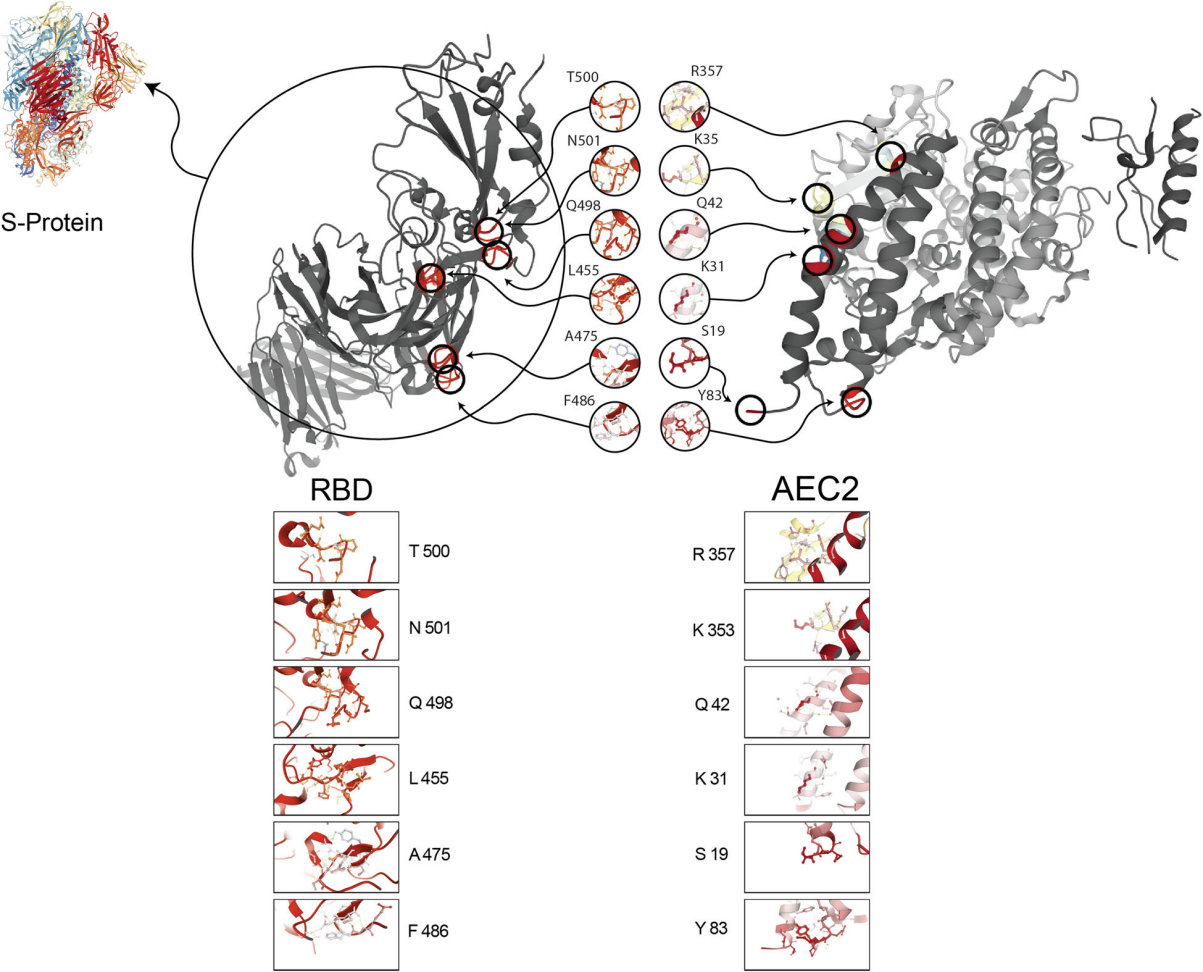
Cartoon representation of the interaction between SARS-CoV-2 S-protein RBD and ACE2. Positions and names of the hot spot residues of the SARS-CoV-2 S-protein RBD/ACE2 are shown. The hydrogen bonds between intra-protein residues are shown by yellow dotted line. The Protein Data Bank (PDB) code for SARS-CoV-2 S-protein is 6VXX, PDB code for RBD region is 7C01 and PDB code for ACE2 is 1R42

The S-protein interactions with ACE2 receptors mediate viral entry whose binding affinity is related to structural parameters like number of hydrogen bonds, interface residues, electrostatic interactions, fraction of polar and nonpolar surface-seeking amino acids, Van der Waals interactions, allosteric effects, and conformational changes [20]. Boosted binding affinity between S-protein and ACE2 was suggested to correlate with increased virus transmissibility and disease severity in humans compared to SARS-CoV [21]. To elucidate why SARS-CoV-2 and SARS-CoV transmissibility is different, first studies have focused on the genome sequence analysis. Studying on full-length genome sequences of SARS-CoV-2 at an early stage of the outbreak confirmed the 79.6% sequence identity to SARS-CoV. ACE2 was recognized as a cellular entry receptor for SARS-CoV-2 like SARS-CoV [22]. Jaimes et al. [23] showed the overall protein sequence identity of the S1 RBD in SARS-CoV-2 and the SARS-CoV was 64%. Interestingly, in the region of S1 containing residues that were shown to directly contact the ACE2 receptor, the common identity between SARS-CoV-2 and SARS-CoV drops to 50%. Despite these differences at the amino acid level, the S-proteins shared a similar folding pattern in both viruses. As expected, at the RBD regions, with most of the amino acid variations, the folding patterns were different. The role of this flexible loop at the RBD region in SARS-CoV-2 would most likely influence on S-protein/ACE2 affinity that results in different viral spreading and pathogenesis. The RBD of SARS-CoV S-protein, located between 318 and 510 residues, binds ACE2 with nanomolar affinity and the receptor-binding motif (RBM) contains four residues (Leu 472, ASn 479, and Thr 482) that are in direct contact with ACE2 located at 424–494 [24, 25] while the RBD of SARS-CoV-2 S-protein, located between 331 and 524 residues and the RBM contains F486, Y489, Q493, G496, T500, and N501 hot spots interacted with ACE2 [26]. In Fig. 1, the RBD/ACE2 interactions were illustrated through PDB code: 6VXX (Closed SARS-CoV-2 S-protein), and PDB code: 1R42 (human ACE2) [27]. Based on surface plasmon resonance (SPR) measurements, the binding affinity between RBD region in SARS-CoV-2 and ACE2 is higher than the SARS-CoV and ACE2 binding affinity [8]. The difference between affinities is related to the different structural features that change type of bonds between RBD and ACE2 [19]. Shang et al. [21] described two main structural differences between SARS-CoV-2 and SARS-CoV interface with ACE2. First, changes in chemical binding because of residues differences: at the RBD and ACE2-binding interface, the salt bridge strength between Lys31 and Glu35 in ACE2 is diminished by distancing and each of the residues forms a hydrogen bond with nearby Gln493 residues from RBD. Replacing Leu472 with Phe486 (with hydrophobic side chain) in SARS-CoV-2, result in stronger contact with the hydrophobic pocket at ACE2. Second, the conformational changes include Gly482, Val483, Glu484, and Gly485 in the RBM of SARS-CoV-2 unite a ridge to become more compact and through this structure connect to the N-terminal helix of ACE2.

## Necessity of thermodynamic assessment in virus-cell interactions

Thermodynamic studies in the biological environment have become progressively important in understanding the principles governing physiological function in the living organism. Protein-protein, protein-ligand, antibody-antigen, protein-nucleic acid, protein-drug interactions, and protein folding/unfolding are part of these physiological functions [28]. According to the nature of the coronavirus surface glycoproteins and cell receptor, to be dissected in more detail in the next sections, thermodynamics of protein-protein interactions can be generalized about virus-cell interactions. If the glycoprotein is taken as a system, each conformational state in the way to absorb to the cell receptor can be considered as a distinct energy state. In this paper, we consider our system composed of the glycoprotein and the surrounded aqueous media with a constant temperature heat and pressure. In this regard, the adsorption on receptors is a well-defined and time-dependent occurring in microseconds and longer timeframes [29].

Specificity and binding affinity are two main determining factors that regulate virus-cell recognition and fusion [30, 31]. Specificity is defined as the ability of a virus to bind a specific receptor. Anchor residues or hot spots contribute significantly to virus-receptor recognition. Since there is a linear relationship between the change in enthalpy and entropy, the favorable changes in entropy are rewarded by the unfavorable change in enthalpy or vice-versa. The virus-cell interactions can be viewed as a reversible process whose strength is defined as binding affinity [32]. The physico-chemical thermodynamic term of binding affinity is translated into the dissociation constant (K_d_) and the physical thermodynamic term of it is translated into the Gibbs free energy of dissociation (ΔG_d_) [33]. Influencing the specificity and binding affinity is the key to influence the virus-cell interactions. Specificity has been the focus of the pharmaceutics studies and subject of the structure-based drug designed [34]. On the other hand, the interaction of the virus with the receptor can be driven by affinity, and changes in enthalpy and entropy.

The nature of this interactions includes electrostatic and non-covalent binding. The non-covalent bonds like hydrogen bonds, salt bridge, and hydrophobic interactions advance recognition and adsorption processes of the virus through enthalpic and entropic changes [35]. Binding free energy, which is composed of enthalpic and entropic terms, can be the subject of investigations to impact molecular recognition and interactions. Therefore, discussion about thermodynamic potentials and kinetics of the SARS-CoV-2 S-protein in its interactions with the ACE2 receptors of the host cell would be extremely valuable for a better understanding of virus virulence.

## Thermodynamic of S-protein adsorption to ACE2

It is generally believed that kinetics and thermodynamics influence the virus interactions with receptors of the host cells. Kinetics controls the rate that a virus comes close to the host cell while thermodynamics potentials can describe how a virus adsorbs into a host receptor [36]. Intermolecular forces, ionic or electrostatic interactions, surface energy, and hydrophobicity are the key parameters in virus adsorption [19, 37]. The fundamental principles of the thermodynamics in all interactions originate from the energy level of the reactants. This section will present an overview of the thermodynamic driving forces that influence the way that S-protein of SARS-COV-2 adsorbs to ACE2 of the host cell.

It assumes that basic thermodynamic potentials like enthalpy and entropy properties of the SARS-CoV-2 S-protein affect the Gibbs free energy of virus-cell interaction and cause the virus protein adsorption. The structural configuration of S1 protein and the types of biochemical and biophysical interactions among its amino acid residues stabilize the protein’s native structure [38]. In other words, the type of amino acids and their chemical characteristic (polar, nonpolar, or ionic charge) by the structure of side chain (R-group) besides intra-protein interactions determine the thermodynamic potentials [14]. These parameters can be influenced by chemical, physical, structural and conformational changes of S protein in adsorption procedure. According to the thermodynamics, S-protein spontaneously adsorb to the ACE2 receptor if the process results in a decrease in the Gibbs free energy of the overall system. The change in the Gibbs free energy is expressed as:

1 $$ \Delta \mathrm{G}=\Delta \mathrm{H}-\mathrm{T}\Delta \mathrm{S} $$

where ΔG is the change in Gibbs free energy of the process under constant temperature and pressure, ΔH is the change in enthalpy, T is the absolute temperature and ΔS is the change in entropy [39]. The amino acid sequence of the S-protein controls its potential energy content base on the structural binding energy. For processes under constant pressure, change in enthalpy is equal to the change in internal energy (ΔU) of the system, bond energy in this specific situation, plus the pressure-volume work (pΔV) done by the system on its surrounding. The change in enthalpy is expressed as:

2 $$ \Delta \mathrm{H}=\Delta \mathrm{U}+\mathrm{p}\Delta \mathrm{V} $$

where ΔU is the change in bond energy, p is the absolute pressure, and ΔV is the change in volume. In the in vivo environment macromolecules are considered as an open, isothermal system at constant pressure and the change in volume for S-protein during adsorption can be considered insignificant; thus, change in enthalpy embodies the change in overall bond energy that occurs during protein adsorption [40]. In the process of S-protein adsorption to the ACE2 receptor, change in bond energy and entropy synchronizes to determine the overall change in the Gibbs free energy. The overall change in the S-protein adsorption enthalpy represents the sum of the changes in bond energy within the S-protein. These changes induced by configuration changes of the S-protein result in changing in biophysical and biochemical interactions in S-protein and subsequently changing in biophysical and biochemical interactions with ACE2 [41]. Similarly, the overall change in entropy (ΔS) represents not only the configuration changes of S-protein as it adsorbs, but also changes in the structure of the receptor protein as well as the other components of the solution. The factors that influence ΔG, ΔH, and ΔS in S-protein binding to the ACE2 receptor are complex and are dependent on several variables such as the amino acids biophysical and biochemical interactions and structural dynamic in surrounding environment. Despite these complexities, basic thermodynamic potentials are relatively straightforward and can be applied to provide a general understanding of making change in binding free energy of the virus-cell system [42].

**Fig. 2:**
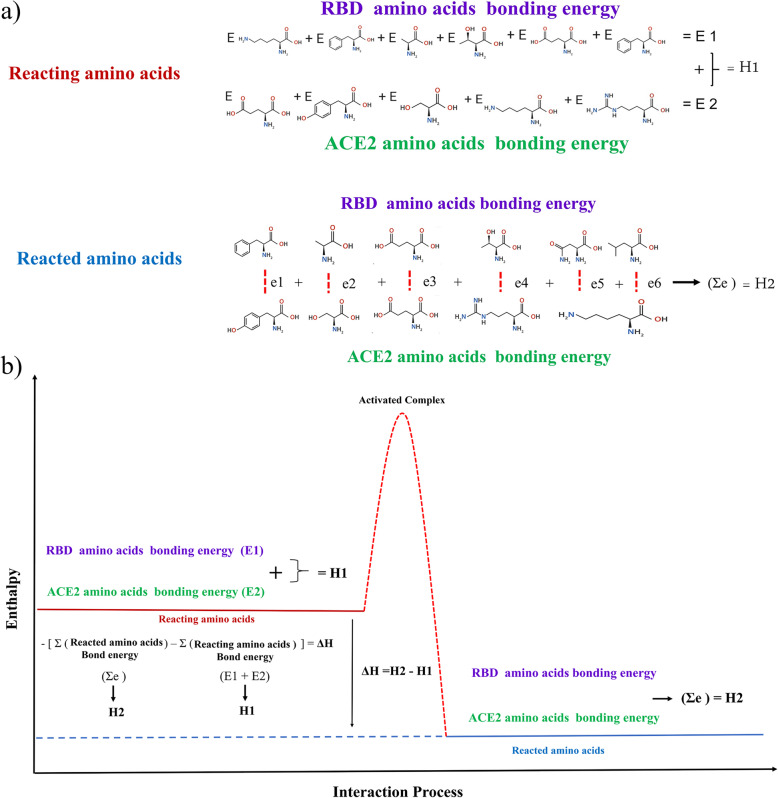
The changes in enthalpy of the SARS-CoV-2/ACE2 host cell system on the association. H1 represents the total reacting amino acids bond energy by adding RBD amino acid binding energies (E1) to ACE2 amino acid binding energies (E2). H2 represents the total reacted amino acids bond energy by adding binding energy of each RBD amino acid to ACE2 amino acid (e1 + e2 + … = Ʃe). Negative values of ΔH indicate the higher potential binding energy level of reacting amino acids (H1 > H2)

Binding of the S-protein to ACE2 needs a certain level of affinity. As mentioned earlier, binding affinity are related to structural-based parameters like number of hydrogen bonds, interface residues, electrostatic interactions, fraction of polar and nonpolar surface amino acids, Van der Waals interactions, and conformational changes [43]. These characteristics directly influence the thermodynamics potentials like dissociation constant, binding free energy, and enthalpy. Binding energy of RBD from SARS-CoV-2 interacting with ACE2 is converted to dissociation constant (K_d_), which has been measured by the SPR for RBD binding to ACE2. According to Wrapp et al. [6], the SPR sensorgram technique showed the SARS-CoV-2 bound ACE2 with around 15 nM affinity which is 10-to-20 fold higher than SARS-CoV binding to ACE2. For the binding of RBD into ACE2 in the body environment, the binding affinity presented as molar Gibbs free energy and relate to the K_d_ via Eq. (3). Furthermore, free-energy of dissociation (ΔG_d_) describes all the chemical and physical energetic factors involved in dissociations reaction and in the physical term, binding affinity can be translated into the ΔG_d_ and free-energy of association (ΔG_a_) through Eqs. (4) and (5) [33]:

3 $$ {\Delta \mathrm{G}}_d=-\mathrm{RT}\ \ln \frac{\mathrm{Kd}}{{\mathrm{c}}^{\uptheta}} $$

4 $$ \Delta  {\mathrm{G}}_{\mathrm{d}}={\Delta \mathrm{H}}_{\mathrm{d}}-\mathrm{T}\Delta  {\mathrm{S}}_{\mathrm{d}} $$

5 $$ {\Delta \mathrm{G}}_{\mathrm{d}}=-{\Delta \mathrm{G}}_{\mathrm{a}} $$

where R is the ideal gas constant, T is the temperature, the c^θ^ = 1 mol/L is the standard reference concentration. Since RBD binging to ACE2 involves several separating and combining different biophysical and biochemical interactions, we can hypothesis that if the spontaneous reaction is favorable exchanging of the charges in these interactions should not be equal to result in the overall change in binding free energy (ΔG < 0). In addition, S-proteins like other active functional proteins fold into specific three-dimensional structures; thus, its native state is metastable. Accordingly, the large kinetic barriers exist between the folded functional states and steady-state which prevent transition into a more stable state. As the virus approaches the host cell, binding free energy of the process of S-protein binding to ACE2 must overcome this kinetic barrier. On the other hand, this metastability facilitates adhesion of the virus into the receptor and conformational changes to a steady-state. Equation (4) indicates that ΔG_d_ upon binding has two components including enthalpic and entropic changes. It also demonstrated that upon binding a favorable enthalpic change should be compensated by an entropic penalty. Predicting the changes in enthalpy and heat capacities along with adhesion of the virus into the receptor is too complicated [44]. Electrostatic interactions, van der Waals interactions, hydrogen bonding, and salt bridge interactions within binding incorporate into enthalpic changes. Hot spots residues in the RBD region have the potential to generate substantial binding energy and a substantially concave topology that cradles the N-terminal helix of ACE2. Residues F486, Y489, Q493, G496, T500, and N501 form the hot spots of the interface with ACE2 protein [26]. Through Fig. 2, the enthalpic changes were explained. H1 represents the energy level of the hot spot residues in the SARS-CoV-2 S-protein RBD region (E1) and energy level of counter residues at ACE2 (E2). H2 represents the predicted total residue binding energy by adding the binding energy of interacting residues at RBD and ACE2 (e1 + e2 + … = Ʃe). The negative values of ΔH indicate the higher potential binding energy level of reacting amino acids (H1 > H2).

We have attempted to address these main potentials in the following sections by addressing some of the primary factors that influence ΔH and ΔS in the SARS-COV-2 S-protein adsorption procedure. Several attempts have been made to describe in more details the biophysical and biochemical characteristics of S-protein and ACE2. We used these details to discuss the energy level of S-protein and the following energy level changes in the process of S-protein adsorption into ACE2.

### Role of conformational changes

When the S-protein binds to ACE2, water molecules that were previously present at the binding site must be displaced. Nonpolar amino acids like Tyr, Phe, and Trp on the surface of the ACE2 are not able to make hydrogen bond effectively with solvent [45]. When the RBD region with its hydrophobic surface interfaces with aromatic residues of these amino acids, the water molecules are released [46]. This hydrophobic effect increases the entropy of the system which called desolvation entropy (ΔS_solv_). The role of protein conformational entropy on binding free energy can be described:

6 $$ \Delta {\mathrm{G}}_{\mathrm{bind}}=\Delta {\mathrm{H}}_{\mathrm{bind}}-\mathrm{T}\left(\Delta {\mathrm{S}}_{\mathrm{protein}}+\Delta {\mathrm{S}}_{\mathrm{receptor}}+\Delta {\mathrm{S}}_{\mathrm{solv}}\right) $$

7 $$ \Delta {\mathrm{S}}_{\mathrm{protein}}=\Delta {\mathrm{S}}_{\mathrm{conf}}+\Delta {\mathrm{S}}_{\mathrm{r}/\mathrm{t}} $$

where ΔS_protein_ is the entropic influence of the S-protein that includes changes in its internal conformational entropy (ΔS_conf_) and changes in the rotational and translational entropy (ΔS_r/t_) [47, 48]. According to the empirical measurement of total entropy changes in protein-ligand interaction by Wand et al. [49], conformational entropy changes have a significant effect on protein function and energetics changes. The significant structural difference between SARS-CoV-2 and SARS-CoV RBMs ridge loop is the presence of four-residue motif Glu-Val-Glu-Gly in SARS-CoV-2 instead of three residue motif Pro-Pro-Ala in SARS-CoV [21]. The limited conformational range of proline residues lowers the conformational entropy of the S-protein in SARS-CoV relative to SARS-CoV-2. The RBD employs conformational entropy in progressing toward the optimum free energy of binding [49]. Lan et al. [50] used X-ray crystallography to determine the structure of the SARS-CoV-2 S-protein RBD-ACE2 complex. The SARS-CoV-2 S-protein RBD core has a five stranded anti-parallel β-sheet and the extended loop has a two short-stranded β-sheet. The extended loop, aka RBM, presents a gently concave surface that cradles the N-terminal helix of ACE2. The overall structure of SARS-CoV-2 S-protein RBD is highly similar to SARS-CoV S-protein RBD; although, an obvious conformational difference has been reported. In another study, it was revealed that RBD in both SARS-CoV-2 and SARS-CoV can adopt either an up or a down conformations. But, the up conformation characteristic of the RBD in SARS-CoV-2 and SARS-CoV are different [9]. These subtle differences between SARS-CoV-2 and SARS-CoV conformational dynamic may contribute to their difference in free-binding energy. On the other hand, the structural studies of S-protein revealed the conformational transitions from receptor-inaccessible state to receptor-accessible state in the receptor binding process [6]. Overcoming the energy barrier for the conformational transition of SARS-CoV-2 S-protein facilitates the adhesion of SARS-CoV-2 to ACE2 and the following entry pathway. The significant variation in free-binding energy of SARS-CoV-2 may have a change in virus binding capacity into the ACE2 receptors and following pathogenicity. Further experiments should clarify the role of thermodynamics of these conformational changes in virus adhesion and membrane fusion.

### Role of glycosylation

Glycosylation is a post-translational modification by which a glycan group covalently attached to a target amino acid residue. This modification strongly influences dynamic conformation, stability, and function of the glycoproteins. Because of the great diversity in monosaccharide building blocks, glycans are highly varied in structure and composition [51]. The CoV S-protein is a heavily N-linked glycosylated, which can be predicted by the sequon Asn-X-Ser/Thr where X is any amino acids except the proline [52]. According to Kumar et al. [53], SARS-CoV-2 interacts with ACE2 receptor using novel glycosylation sites relative to SARS-CoV. They have suggested that different glycosylation sites may result in different viral fusion and associated viral spreading and pathogenesis. 13 out of the 22 N-linked glycosylation sites in SARS-CoV-2 S-protein are in the S2 subunit and 9 of them are located in S1 subunit. According to site-specific mass spectroscopy analysis, there is a large population of complex-type glycans displayed on SARS-CoV-2 S protomer relative to oligomannose-type and hybrid-type glycans which results in less densely glycosylated protein in comparison with S-protein in HIV-1 Env which contains about 60% oligomannose-type glycans. Oligomannose-type glycans are protected by the protein component while complex-type glycans are located on the extended loop structure [13]. Table 1 summarizes the averaged compositions across all glycosylation sites of the SARS-CoV-2 S-protein and their schematic structures.

**Table 1:**
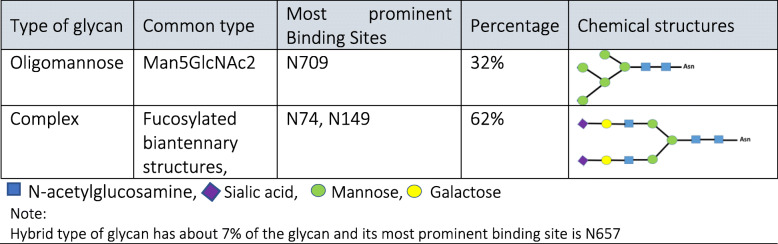
Averaged compositions across all glycosylation sites of the SARS-CoV-2 S-protein and their schematic structures

Effect of glycosylation on biophysical characteristics of the proteins like structure, folding, solubility, enzyme resistance, thermal and kinetic stability, and aggregation has been reported in some studies [54] . However, role of glycosylation in thermodynamic stability is controversial. In vitro loss of stability of proteins reported upon glycosylation in various studies may be correlated to conformational distortions to their native state caused by interactions between sugar and the protein surface [55]. Gavrilov et al. [55] studied the effect of glycosylation on the stability of native state of the MM1 protein. They claimed that the attached glycans may intensely interact with the protein surface, which results in changing the intra-protein energy interactions but the destabilization in the folded state is not the result of losing these interactions. They assumed that glycosylation may change the enthalpy of the unfolded state by shifting the balance between the short-range and long-range glycan-protein interactions. Protein dynamics is another feature that is affected by glycosylation. Lee et al. [56] measured the fluctuations of each residue during glycosylation through atomic molecular dynamics simulation. The results showed that the impact of glycosylation is not restricted to the residues near the glycosylated sites and can be propagated to other regions of the protein; as a result, the potential entropy loss would be minimized. They also claimed that glycosylation decreases protein dynamics and stabilize the protein structure. In contrast, it has been also reported that glycosylation might reduce the thermodynamic stability of the tyrosinase family protein [57].

For comparison between glycosylation sites of RBD region of SARS-CoV-2 and SARS-CoV, we used the S-protein sequence of SARS-CoV-2 from GenBank accession numbers QHR63250.1 and AY278488.2 for SARS-CoV. The predicted glycosylation sequences were determined by NetNGlyc 1.0 software (http://www.cbs.dtu.dk/services/NetNGlyc/) [58]. In Table 2, predicted glycosylation sequences were listed and asparagines within ASn-X-Ser/Thr sequons were colored purple with potential of N-glycosylation in vivo [53]. Differences in glycosylation sites between SARS-CoV-2 and SARS-CoV result from sequence variation. Earlier, it was proved that the degree and location of glycosylation influence on biophysical properties of proteins [54]. Glycosylation sites are the place of new interactions between glycans and amino acids that have a stabilization effect. The enthalpic or entropic origin of this effect is controversial. More glycans attached to the protein lead to a decrease in the entropy and higher free-energy barrier. In SARS-CoV-2 S-protein, the overall glycosylation sites are less than SARS-CoV with fewer glycosylation sites in the RBD region (Table 2). We can hypothesize that SARS-CoV-2 S-protein has a lower entropy and stabilization, therefore; it shows a lower free-energy barrier. Furthermore, carbohydrate hydroxyls at glaycans replace several of the protein surface water interactions so reduces the tendency for aggregation and increased solubility. On the other hand, the presence of glycans poses a steric hindrance to viral attachment to the receptors [59]. As a result, a smaller number of glycosylation sites at the RBD regions could ensure high-affinity binding. Through Table 2, the predicted glycosylation sites were compared between SARS-CoV and SARS-CoV-2 in which predicted glycosylation sites are lower in SARS-CoV-2.

**Table 2:**
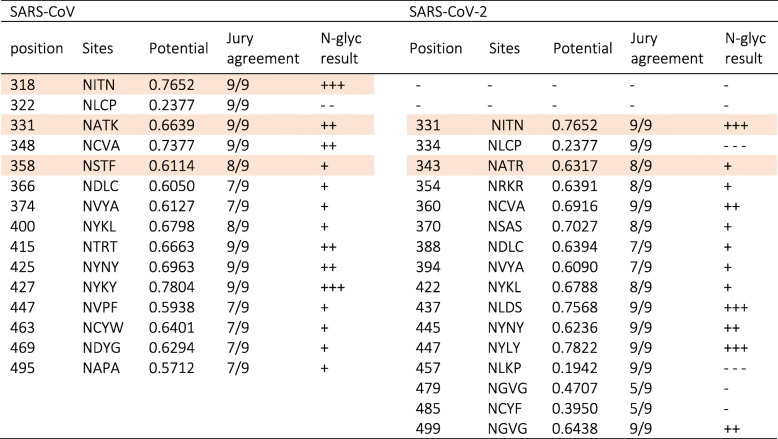
List of the predicted glycosylation sequences and the potential N-glycosylated sites in vivo were colored purple. GenBank accession numbers QHR63250.1 (SARS-CoV-2 S), AY278488.2 (SARS-CoV S)

### Role of polarity and hydrophobicity

**Fig. 3:**
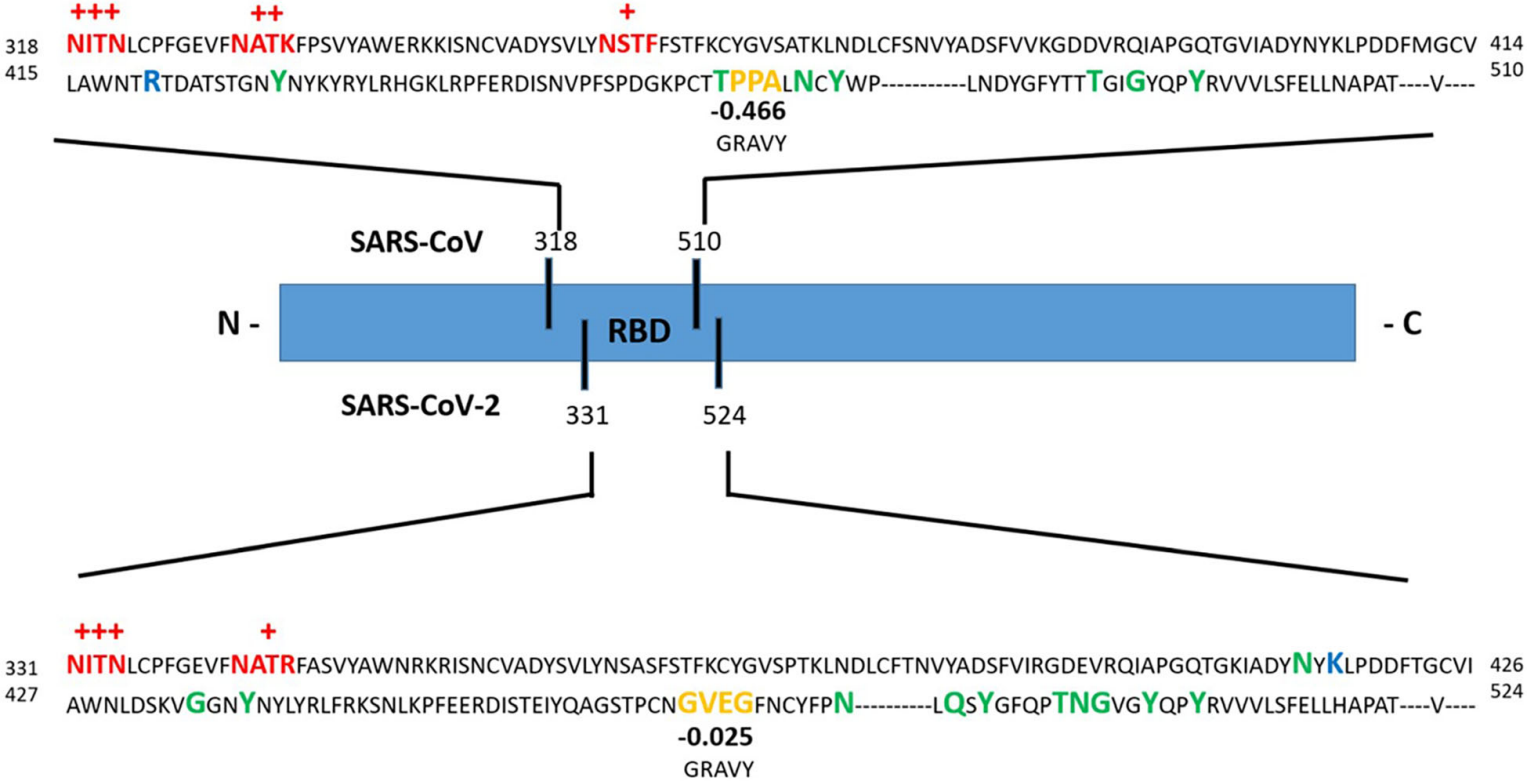
Compering the sequence differences between SARS-CoV-2 and SARS-CoV S-protein RBD regions. Red sequon represented the potential in vivo glycosylation sites, green residues represented the place of hydrogen bonds, and blue residues participating in the salt-bridge. The two important sequon were colored in orange with their GRAVY number. GenBank accession numbers QHR63250.1 (SARS-CoV-2 S), AY278488.2 (SARS-CoV S)

The physiological dielectric constant is one of the important parameters influencing virus adsorption to host cells due to its effect on intermolecular forces [60]. The dielectric constant of the bulk water is about 80 units and the actual value of the dielectric constant of proteins are still a continuing argument, but it is considered to be much lower than bulk water [61]. The strength of the electrical double layer formed around the virus and the cell receptors depends on the dielectric constant of their residues. Polar, nonpolar, and charged residues have a different dielectric constant in the physiological solution. Generally, hydrophilic polar and charged residues besides glycosylation sites forming the outer surface of the protein support water solubility while hydrophobic residues present at the outer surface participate in the hydrophobic interactions of virus/receptor adsorption [62]. The virus near the host cells can be considered as an independent heterogeneous particle consisting of segments with various biochemical properties, and the virus adsorption can only occur when the energy gain of virus/receptor outweighs the receptor/solvent interactions. Prabakaran et al. [59] analyzed the ACE2 structure 3D model showed at the top of the ACE2 there was a negatively-charged ridge surrounded by the hydrophobic patches. This deep channel complements the positive charges of the RBD loop and the hydrophobic patches at the ACE2 interact with hydrophobic residues of the RBD loop. Besides, networks of hydrophilic interactions were identified at the RBD/ACE2 interface. Lan et al. [50] indicated 13 hydrogen bonds and 2 salt bridges at SARS-CoV-2 RBD/ACE2 interface. In Fig. 3, the residues participating in hydrogen bonds in the salt-bridge were shown. Residues participating in these hydrophilic networks are different in SARS-CoV-2 and SARS-CoV S-protein RBD regions. The diversity of the residues participating in SARS-CoV-2 was more than SARS-CoV.

Studying the structural and dynamical differences among the coronavirus could explain the boosted transmissibility of SARS-CoV-2. Li et al. [25] showed a key factor determining severity and human-to-human transmissibility of SARS-CoV relative to civet viral strains is the presence of a γ–methyl group on Thr487 side chain. Generally, nonpolar residues cause disruption of highly dynamic hydrogen bonding network between water molecules; thus, the mobility of the residues and water molecules are strongly limited. In terms of thermodynamics, this mobility restriction causes significant losses in translational and rotational entropy of residues and water molecules which is unfavorable in terms of free-energy of the system. In some studies, potential residues involved in the RBD and ACE2 interactions were identified through computer modeling and X-ray crystallography [63]. In SARS-CoV-2, Asp with polar amine group is replaced with Thr. Asp hydropathy index is − 3.5 while the Thr hydropathy index is − 0.7, indicating increased hydrophobicity. In another example, Leu486 with hydropathy of 3.8 in SARS-CoV is replaced by Phe with 2.8 hydropathy in SARS-CoV-2. These changes in hydrophobicity of the residues result in reducing translational and rotational entropy. On one hand, the presence of four-residue motif Glu-Val-Glu-Gly in SARS-CoV-2 instead of three-residue motif Pro-Pro-Ala in SARS-CoV has a significant effect on the grand average hydrophobicity (GRAVY). GRAVY calculates the sum of the hydropathy values of the selected region and divided by the selected sequence length (Kyte and Doolittle method) [64]. The GRAVY of Glu-Val-Glu-Gly in SARS-CoV-2 is − 0.025 and it is more positive than the GRAVY in SARS-CoV which is − 0.466 (more hydrophobic). The hydrophobic effect can also be described as the positive free-energy change of the residue and decomposed into enthalpic and entropic charities. These findings reveal that even one or a few amino acid residue variations in viral RBDs can change binding free-energy through hydrophobic contribution. On the other hand, lysine hotspots (Lys31 and Lys353) on ACE2 are critical for coronavirus RBDs binding because the positively-charged and hydrophobic residues of Lys need to be neutralized [65]. It can be hypothesized that SARS-CoV-2 with more positive GRAVY in the RBD region recognizes ACE2 better that SARS-CoV S-protein RBD. Although, GRAVY calculation of RBD loop at SARS-CoV and SARS-CoV-2 revealed that their GRAVY is in a similar rang (− 0.202 in SARS-CoV-2 in comparison with −.0236 in SARS-CoV).

## Concluding remarks and perspectives

The nature and structure of the SARS-CoV-2 S-protein and its interactions with the ACE2 have been the subject of many studies. It is worth expanding thermodynamic analysis to these interactions. Thermodynamic studies in the biological environment have become progressively important in understanding the principles governing physiological functions. Generally, understanding and regulating virus/cell recognition and interactions are part of the pharmaceutics studies. Therefore, these assessments would be tremendously valuable in the design of candidate pharmaceutical agents or alternative solutions. This study has probed the interaction between SARS-CoV-2 and host cell receptors by emphasizing the governing kinetic and thermodynamic rules. In the process of developing an in-depth understanding of entropy and enthalpy changes in the context of this interaction, we addressed some of the crucial factors related to viral and receptors structures that are the principal of this adsorption procedure. The spontaneous adsorption (ΔG < 0) of the S-protein in SARS-CoV-2 indicates that the adsorption process must be driven by a decrease in enthalpy (H1 > H2) and an increase in entropy (S1 < S2). Unlike ΔH which is based on molecular contacts at the binding interface, ΔS involves changes in structure and mobility. S-protein of SARS-CoV-2 needs conformational and rotational changes to expose the engaging “up” positions of its RBD at reacting site that is interpreted to conformational entropy which is part of the total entropy changes. Given these findings, displacing any existing molecules at the binding site decreases the energy level of S-protein (H1) and can lead ΔG to positive values. On the other hand, physical inhibitors that force more dynamic configurations on S-protein alter the energy level of the S-protein and make ΔS < 0 or ΔG > 0. Another important issue to underline is that the presence of the glycosylation sites that serve as a barrier to viral attachment to ACE2. In SARS-CoV-2 S-protein, the overall glycosylation sites are less than SARS-CoV which results in lower entropy and stabilization of SARS-CoV-2 with a lower free energy barrier to binding. On the other hand, the hydrophobic effect can also be described as the positive free-energy change of the residues and decomposed into enthalpic and entropic characters. These findings reveal that even one or a few amino acid residues in viral RBDs can change binding free energy through hydrophobic contribution. Polar, nonpolar, and charged residues have a different dielectric constant in physiological milieu. Generally, hydrophilic polar and charged residues beside glycosylation sites form the outer surface of the protein to provide water solubility, while hydrophobic residues present at the outer surface participate in the hydrophobic interactions of virus/receptor adsorption. Binding free-energy change is related to several structure-based factors, such as conformational features, hot spots, and number of hydrogen bonds. In this regard, any stimulation in surrounding molecular bonds alters the electron clouds around the amino acid atoms might alter the thermodynamic balance and stops the spontaneous interactions between the S-protein and the cell. Accordingly, physical inhibitors might have the potential to hinder the interaction between SARS-CoV-2 virus and host cell receptors. Ultimately, the fact that increasing the temperature deducts the bound sites is an essential step toward considering kinetic parameters like pH and temperature at the reacting point like other thermodynamic inhibiting parameters to stop the viral diffusion.

## Data Availability

The coordinates and structure factor files for the Closed SARS-CoV-2 S-protein, RBD region, and hACE2 have been deposited in the Protein Data Bank (PBD) with accession cods 6VXX, 7C01, and 1R42 subsequently. The S-protein sequence of SARS-CoV-2 and SARS-CoV analyzed in this study are available at GenBank accession numbers QHR63250.1 (SARS-CoV-2 S), AY278488.2 (SARS-CoV S). The predicted glycosylation sequences were determined by NetNGlyc 1.0 software (http://www.cbs.dtu.dk/services/NetNGlyc/). GRAVY values were calculated through a sequence-based predictions website (http://www.gravy-calculator.de/index.php).

## Abbreviations

ACE2: Angiotensin Converting Enzyme 2
COVID-19: Coronavirus Disease 2019
RBD: Receptor Binding Domain
RBM: Receptor Binding Motif
SARS-CoV-2: Severe Acute Respiratory Syndrome Coronavirus 2
SARS-CoV: Severe Acute Respiratory Syndrome Coronavirus
SPR: Surface Plasmon Resonance
